# Take a “Selfie”: Examining How Leaders Emerge From Leader Self-Awareness, Self-Leadership, and Self-Efficacy

**DOI:** 10.3389/fpsyg.2021.635085

**Published:** 2021-03-25

**Authors:** Eva M. Bracht, Fong T. Keng-Highberger, Bruce J. Avolio, Yiming Huang

**Affiliations:** ^1^Department of Social Psychology, Institute of Psychology, Goethe University, Frankfurt, Germany; ^2^Nanyang Business School, Nanyang Technological University, Singapore, Singapore; ^3^Foster School of Business, University of Washington, Seattle, WA, United States; ^4^School of Business, Nanjing University, Nanjing, China

**Keywords:** information processing theory, leadership emergence, leader self-awareness, leader self-efficacy, self-leadership, social cognitive theory

## Abstract

It is important to understand the processes behind how and why individuals emerge as leaders, so that the best and most capable individuals may occupy leadership positions. So far, most literature in this area has focused on individual characteristics, such as personality or cognitive ability. While interactions between individuals and context do get research attention, we still lack a comprehensive understanding of how the social context at work may help individuals to emerge as leaders. Such knowledge could make an important contribution toward getting the most capable, rather than the most dominant or narcissistic individuals, into leadership positions. In the present work, we contribute toward closing this gap by testing a mediation chain linking a leader's leader self-awareness to a follower's leadership emergence with two time-lagged studies (*n*_study1_ = 449, *n*_study2_ = 355). We found that the leader's leader self-awareness was positively related to (a) the follower's leadership emergence and (b) the follower's nomination for promotion and that both relationships were serially mediated by the follower's self-leadership and the follower's leader self-efficacy. We critically discuss our findings and provide ideas for future research.

## Introduction

Who emerges as a leader and how qualified are they to lead? These questions have long been discussed in the research and practice literature on leadership, given the importance that leaders play in all aspects of society. Thus far, the literature on antecedents of leader emergence, which is the degree to which an individual is perceived by others as being a leader (Judge et al., 2002), has largely focused on individual attributes. More precisely, personality factors like agreeableness (Wyatt and Silvester, 2018) and extraversion (Reichard et al., 2011), as well as dominance (Hegstrom and Griffith, 1992) and narcissism (Nevicka et al., 2011), have been shown to be relevant to predicting leadership emergence. Moreover, knowledge and skills, in terms of emotional awareness/recognition (Walter et al., 2012) and communication (Charlier et al., 2016), as well as identity-related factors, such as leader role identity (Kwok et al., 2018), or leader self-efficacy (Liu et al., 2019), have also been shown to play vital roles in predicting the emergence of leaders.

In our research, we want to shift the focus in examining leader emergence to the context, as recommended by Avolio (2007), because “one can learn most about individual behavior by studying the informational and social environment within which that behavior occurs and to which it adapts” (Salancik and Pfeffer, 1978, p. 226). Recent work on the intersection of the individual and their social environment in predicting leader(ship) emergence has included a focus on the role of status (McClean et al., 2018), peer liking (Hu et al., 2019), and leader–member exchange (Zhang et al., 2012). Additional work on antecedents of leadership emergence has shown that network centrality can also play a role in predicting who emerges as a leader (Kwok et al., 2018). While prior work has provided a preliminary foundation for understanding the individual and relational antecedents for leadership emergence, this work does not yet explain how an individual's social environment at work influences their development and whether those developmental gains predict leader emergence.

**Figure 1:**
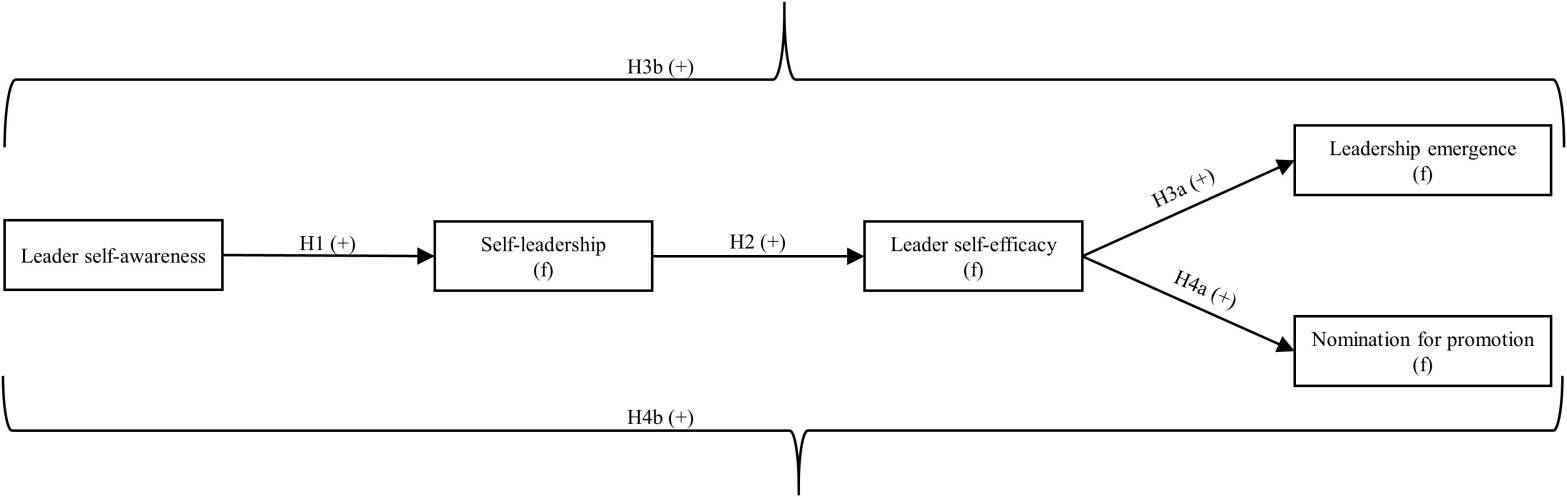
Theoretical model. (f), follower-related variable.

Contributing toward closing this gap, and following the call from Acton et al. (2019) to understand leadership emergence as a dynamic, interactive process, we explore in two field studies how a leader's inner, self-developmental leadership process can lead to a follower's emergence as a leader through its effect on a follower's leadership development process. We theorize and test how the target leader's leader self-awareness can ultimately lead to (a) the follower's leadership emergence and (b) the follower's nomination for promotion into a leadership position. Specifically, we draw from both social information processing and social cognitive theory to propose how these relationships are both serially mediated by the follower's own self-leadership development and the follower's leader self-efficacy ^1^. Figure 1 summarizes the proposed relationships in this research.

Our main theoretical contribution to leader(ship) emergence literature lies in describing how leadership emergence can result from a developmental process involving individual as well as social context variables at the workplace. We consider this an important contribution, since (a) the leadership emergence literature thus far has largely captured a static perspective on antecedents of leadership emergence, and (b) a process-oriented perspective involving multiple components of individuals and their context is of central importance given that leadership results from interactions between individuals and context (Porter and McLaughlin, 2006; Avolio, 2007; Jepson, 2009; Oc, 2018).

Our work also contributes to practice by examining how leaders can positively influence their followers' leadership emergence. Using their influence to promote leadership emergence in their followers, leaders may actively contribute to the emergence of effective and capable leaders, rather than relying upon the most dominant (Hegstrom and Griffith, 1992) or narcissistic (Nevicka et al., 2011) individuals to emerge as leaders.

Explaining the relationship between leader self-awareness and a follower's self-leadership, we rely on *Social Information Processing Theory* (Salancik and Pfeffer, 1978). Following this theory, individuals adapt their attitudes, behaviors, and beliefs to their social context. This happens in two ways. First, individuals use the cues of their social environment to construct meaning regarding what are acceptable beliefs, attitudes, and behaviors in the particular social context. Second, the social context heightens the salience of certain information and thereby increases its relevance to the individual. In other words, individuals develop their attitudes as a function of the social information that is available to them.

Salancik and Pfeffer (1978) further described that relevant contextual information for attitude and behavior formation at work is, among other factors, a leader's style of supervision. Hence, a leader's behavior and attitude can impact the follower's attitudes, beliefs, and behaviors. These relationships have already been demonstrated in several previous studies (e.g., Groves and LaRocca, 2011; Steinmann et al., 2018; Farahnak et al., 2020). Thereby, the relevant source of information for attitude formation and behavior is not the leader's behavior or attitude itself but an individual's perception. This is because characteristics of an individual's social context are constructed by them, as they navigate through their daily interactions (Salancik and Pfeffer, 1978). In short, a leader's attitudes and behaviors as perceived by their follower can impact the follower's decisions and behavior. Building on this, we can outline the relationship between leader self-awareness and follower self-leadership.

Leader self-awareness has been defined based on previous conceptualizations of self-awareness but linking awareness specifically to how a leader views their leadership of others [see Walumbwa et al. (2008)]. More precisely, leader self-awareness refers to leaders “demonstrating an understanding of how one derives and makes meaning of the world and how that meaning making process impacts the way one views himself or herself over time” (Walumbwa et al., 2008, p. 95). Thereby, leader self-awareness can be observed in terms of specific leader self-awareness behaviors a person who leads exhibits, like seeking feedback to improve interactions with others (Avolio et al., 2018).

Self-leadership is defined as the process of influencing oneself to achieve goals (Houghton and Neck, 2002). Individuals influence and lead themselves by using specific sets of cognitive and behavioral strategies (Neck and Houghton, 2006). These strategies are labeled as (1) *behavior focused*, (2) *natural reward*, and (3) *constructive thought patterns*. First, behavior-focused strategies “strive to heighten an individual's self-awareness in order to facilitate behavioral management” (Neck and Houghton, 2006, p. 271). These behavioral strategies include different elements. For example, the self-leadership process may start with *self-observation*, which implies being aware of when and why one engages in specific behaviors, which may lead to the individual identifying goals for change. What may follow then is *self-goal setting*, which refers to setting and working toward specific goals. On the way toward goal achievement, it is important for self-leaders to *self-cue* or keep track of those goals in order to stay motivated, for instance by using lists, notes, or motivational posts [for more detailed descriptions of the strategies, see Houghton and Neck (2002), Neck and Houghton (2006), Stewart et al. (2011), Stewart et al. (2019)].

Having succeeded or failed at reaching a goal, individuals then engage in the second type of strategy known as *natural reward*, which refers to building pleasant and enjoyable features into one's work tasks (Houghton and Neck, 2002; Neck and Houghton, 2006; Stewart et al., 2011, 2019). The third self-leadership strategy, *constructive thought patterns*, refers to evaluating one's beliefs and assumptions, as well as using mental imagery and positive self-talk strategies. Taken together, these strategies describe how individuals gain awareness over their beliefs and behavior and then consciously work toward, and mentally track, the realization of their goals.

We suggest that leader self-awareness, as observed by the follower, may encourage the follower to engage in developing his/her own self-awareness-related aspects of self-leadership, which then motivates self-leadership development. More specifically, followers who observe their leader to be self-aware, should tag self-awareness as being an important attitude and behavior in the respective work context (cf., Salancik and Pfeffer, 1978). Consequentially, followers may feel inspired to heighten their self-awareness as well, which may include those elements of self-leadership that are related to leader self-awareness, such as self-observation, reflecting on and keeping track of their goal achievement and becoming aware of their self-talk and self-imagery. Previous work has provided evidence connecting observed leadership with self-leadership as well. Different from our proposition, these other studies largely focused on empowering leadership (e.g., Amundsen and Martinsen, 2014, 2015) or on the self-awareness component as part of empowering leadership (Tekleab et al., 2008). Building on this, we propose our first hypothesis:

*H1: Leader's leader self-awareness is positively related to follower's self-leadership*.

Beyond being impacted by one's social context, *Social Cognitive Theory* suggests that individuals influence their own attitudes and behaviors themselves (Bandura, 1991). For instance, when individuals repeatedly succeed at attaining their goals, or perform well, they develop positive self-efficacy beliefs (Bandura and Adams, 1977; Sitzmann and Yeo, 2013). Self-efficacy beliefs are defined as “people's beliefs in their capabilities to mobilize the motivation, cognitive resources, and courses of action needed to exercise control over events in their lives” (Wood and Bandura, 1989, p. 364).

When individuals successfully perform a certain skill or behavior, this does not only increase their self-efficacy beliefs regarding the practiced skill or behavior (e.g., Talsma et al., 2018), but it may also encourage individuals to set higher standards and goals for themselves as they move forward in their work (Wood and Bandura, 1989). For instance, a leader who successfully leads a team of five may consequently feel confident enough to lead a larger team next. In other words, success in one area may expand to self-efficacy toward a more complex challenge. Since self-efficacy relates to what the individual has successfully accomplished in previous experiences, a virtuous circle of self-efficacy and performance develops.

Based on the idea of a self-efficacy/performance virtuous cycle, we suggest that successfully performing as a self-leader may strengthen one's confidence to not just lead oneself but to do so with others as well. Thereby, the confidence to lead others can be expressed in the form of leader self-efficacy. Leader self-efficacy is defined as “leaders' beliefs in their perceived capabilities to organize the psychological capabilities, motivation, (…) and courses of action required to attain effective, sustainable performance across their unique leadership roles, demands, and contexts” (Hannah et al., 2012, p. 144). Self-leadership may increase leader self-efficacy for two reasons. First, self-leadership has been defined as the equivalent of leadership, whereby self-leadership is focused on leading *oneself*, while leadership is focused on leading *others* (Furtner, 2017). Hence, self-leadership and leadership can be seen as two constructs that belong generally within the same skill domain. Second, self-leadership has often been considered a precondition for leadership. More precisely, research has argued and shown that self-leadership is a helpful skill in positively contributing to leading others successfully (cf., Drucker, 1999; Lovelace et al., 2007; Furtner et al., 2013). Based on the above arguments, that individuals who successfully lead themselves may feel more confident to lead others, we propose our second hypothesis:

*H2: Follower's self-leadership is positively related to follower's leader self-efficacy*.

Building on social cognitive theory, previous work could show that individuals not only develop self-efficacy beliefs based on their prior performance but that this mechanism can work the other way around as well (Kroesen et al., 2017; Talsma et al., 2018). More precisely, individuals tend to choose those activities they feel self-efficacious about, such that they choose those activities they believe they can execute successfully (Wood and Bandura, 1989). This belief encourages them to exert more effort and direct more persistence toward that task (Bandura, 1988). For example, it has been shown that creative self-efficacy can enhance one's creativity associated with creative ideation (e.g., Yang et al., 2020).

Extending this to leader self-efficacy, followers with high leader self-efficacy should emerge as leaders. We suggest such a relationship based on previous findings showing that leader self-efficacy predicted leadership emergence (Liu et al., 2019). Hence, we propose our Hypothesis 3a:

*H3a: Follower's leader self-efficacy is positively related to follower's leadership emergence*.

Combining these arguments with Hypotheses 1 and 2, we propose the following sequential mediation relationship:

*H3b: The indirect relationship between leader's leader self-awareness and follower's leadership emergence is mediated by follower's self-leadership and follower's self-efficacy*.

Now, completing the theoretical linkages, we suggest that a follower's leadership self-efficacy will not remain unnoticed, improving their chances of being chosen for leadership roles. When individuals feel confident to lead, this may positively impact other people's evaluations of that individual's capability to lead. Such relations have been found in the area of creativity (Gong et al., 2009), whereby an individual's creative self-efficacy was positively related to the same individual's level of creativity, as rated by others.

In a similar vein, leader self-efficacy has been shown to be positively related to other-rated performance and leadership (Hannah et al., 2012). Assuming that leaders do notice their followers' feelings, and displays of confidence in their own leadership, and consider this as being a relevant criterion for promoting a follower into a leadership position, we propose the following hypothesis:

*H4a: The follower's leader self-efficacy is positively related to the follower*'*s nomination for promotion*.

Combining these arguments with Hypotheses 1 and 2, we propose the following sequential mediation relationship:

*H4b: The indirect relationship between leader's leader self-awareness and the follower's nomination for promotion is mediated by follower's self-leadership and follower's self-efficacy*.

## Method Study 1

### Sample

The sample for Study 1 was recruited through the online panel provider called Kantar, which is an international organization based in London. Kantar has access to a participant pool of several million respondents globally. Selection criteria for our study included (a) holding American citizenship, (b) being aged between 18 and 65, (c) being employed, and (d) agreeing to be recontacted for the second half of the study. Kantar elicited our invited participants for this study via an email to the panel, using a short description of our study. Participation in this study was voluntary, and participants were paid a standard fee for completing the investigation. In order to mitigate single source/common method bias, as well as social desirability effects, we used a 4-week interval between Time 1 and Time 2 data collection. At Time 2, a total of 717 participants completed our survey. After having excluded participants who did not fully answer both surveys, did not pass all attention checks (cf., Paas and Morren, 2018), or showed a particular answer pattern like giving extremely positive or negative answers throughout the survey, our sample resulted in a total number of *n* = 449 participants.

In the final sample, participants were 20–65 years old (*M* = 51.33, *SD* = 11.14), and the majority was female (72.2%). Among the participants, 1.3% did not graduate from school, 18.3% completed vocational education, and 20% held a High School degree. Another 34.5% completed their Bachelor's, 12.9% finished their Master's, and a small percentage of 2.4% held a Ph.D. degree. The majority of participants were working in organizations with up to 200 employees. The size of the organization employees worked in, varied from 2 to 2.2 million employees (*M* = 28,965.01, *SD* = 174,747.66). Some individuals had just joined their organizations a few months ago, while others were working in the same organization for up to 43 years (*M* = 12.01, *SD* = 10.72). Participants covered a broad range of industries, from health (14.5%), to the educational sector (12.9%), public services (6.9%), and IT (4.5%). While most individuals did not have leadership experience (66.6%), those who were in leadership positions had up to 10 years of leadership experience (58.4%).

### Measures

In this study, we measured leader self-awareness, self-leadership, and leader self-efficacy as core variables. Thereby, leader self-awareness and self-leadership were measured at Time 1 and leader self-efficacy at Time 2. Furthermore, we included gender as a control variable. We did so because gender has been shown to significantly affect leadership emergence [for a meta-analysis, see Badura et al. (2018)]. Differences in leadership emergence between men and women can, for instance, be traced back to agentic and communal traits (Badura et al., 2018). More precisely, as shown by Badura et al. (2018), women exhibit less agentic traits and, as a consequence, participate less in group discussions, which makes them less likely to emerge as leaders, compared to their male counterparts. Gender differences were not only found with respect to leader and leadership-related variables but also regarding the use of different self-leadership strategies (Bendell et al., 2019).

We measured leader self-awareness with the 4 items from the *Leader Self-Awareness* dimension of the *Authentic Leadership Questionnaire* (Avolio et al., 2018), provided by www.mindgarden.com. A sample item is: “*My leader shows he or she understands how specific actions impact others*.” Cronbach alpha of the scale was α = 0.91. Answers were given on a scale from 1 = *not at all* to 5 = *frequently, if not always*.

Self-leadership was captured using the *Abbreviated Self-Leadership Questionnaire* (Houghton et al., 2012), which is a nine-item measure of self-leadership. A sample item was: “*I try to mentally evaluate the accuracy of my own beliefs about situations I am having problems with.”* Cronbach alpha was α = 0.90. Participants rated the items on a scale from 1 = *strongly disagree* to 5 = *strongly agree*.

Leader self-efficacy was measured with a *Leader Self Efficacy Scale* (Hannah et al., 2012). More precisely, we chose the *Action* dimension of the leader self-efficacy scales because it most adequately represented the kind of leader self-efficacy we were interested in testing. This dimension has seven items, and participants rated their efficacy for exhibiting a certain behavior such as: “*I energize others to achieve their best.”* Cronbach alpha of the scale was α = 0.95. Participants chose a value for each item on a Likert scale between 1 = *strongly disagree* and 7 = *strongly agree*.

### Analysis

We tested our hypotheses using structural equation modeling techniques with Mplus version 8 (Muthén and Muthén, 1998–2017). In our analysis, we let the items for each construct load on the respective latent factor and then modeled the direct and indirect paths between the latent constructs. In this study, we calculated a model testing Hypotheses 1 and 2. To control for gender, we regressed self-leadership and leader self-efficacy on gender.

We examined several indicators of model fit. The chi-square value shows exact model fit and should be insignificant to indicate good model fit (Geiser, 2011). However, the chi-square test is sensitive to large sample sizes, making it good practice to complement the chi-square test with additional goodness-of-fit indicators. First, we used the comparative fit index (CFI), capturing incremental fit with values close to 1, indicating that the model explained the data better than an independence model. Values above 0.90 are suggested to avoid accepting miss-specified models (Hu and Bentler, 1999). Second, the root mean square error of approximation (RMSEA) indicates approximate fit, which should be 0.08 or lower (Browne and Cudeck, 1993). Third, the standardized root mean square residual (SRMR) provides an overall evaluation of the residuals, whereby values close to 0.08 indicate that the observed (co-)variances should be replicable (Hu and Bentler, 1999). Hu and Bentler (1998) suggested that researchers should use a two-index presentation strategy, saying that when the SRMR is close to 0.08 in combination with either CFI (close to 0.95) or RMSEA (close to 0.06), there is a relatively good fit between the model and the data.

## Results Study 1

We calculated means, standard deviations, and correlations for each scale. Results showed that all correlations between our core variables were significant at *p* < 0.01. Specifically, correlations were *r* = 0.38 between leader self-awareness and follower's self-leadership, and *r* = 0.49 for self-leadership and leader self-efficacy. Beyond this, gender correlated significantly with leader self-efficacy (*r* = 0.10, *p* < 0.05), indicating that male participants had higher levels of self-efficacy. Yet, gender did not correlate significantly with any other variable. All results can be found in Table 1.

**Table 1:** Means, standard deviations, and bivariate correlations for Study 1.

	**Mean (SD)**	**1**	**2**	**3**
Leader self-awareness (l)	3.06 (1.13)			
Self-leadership (f)	4.98 (1.25)	0.38^**^		
Leader self-efficacy (f)	4.38 (1.48)	0.28^**^	0.49^**^	
Gender^a^ (f)	–	0.03	0.00	0.10^*^

** *p < 0.01,*

* *p < 0.05.* *(l), leader-related variable; (f), follower-related variable*.

a *Female participants were coded as 1, male participants as 2*.

To determine whether our measures were sufficiently different from each other, we tested them for their discriminant validity. Discriminant validity can be confirmed to the degree that a latent variable explains a higher amount of variance in its indicator variables than it shares variance with other constructs (Fornell and Larcker, 1981). This criterion is met if the average variance extracted (AVE) regarding the focal factor is higher than its *r*^2^ with other factors (Henseler et al., 2015). Based on this criterion, we compared the AVE values of each construct in the model with its squared correlations with the remaining constructs. Results show that the AVE value for leader self-awareness was 0.72, which was higher than its squared correlations with self-leadership (*r*^2^ = 0.16) and leader self-efficacy (*r*^2^ = 0.08). Moreover, the AVE for self-leadership was 0.54 and was higher than its squared correlations with leader self-awareness and leader self-efficacy (*r*^2^ = 0.26). Finally, the AVE for leader self-efficacy was 0.73 and was higher than the squared correlations with leader self-awareness and self-leadership. Hence, we could confirm discriminant validity for all constructs in this study.

We tested for common method variance to help mitigate any systematic bias in our data. To do so, we conducted an exploratory factor analysis, which included all variables used in the model. We can conclude that there was no evidence for common method variance bias in our results when we tested how much variance one overall method factor accounted for in terms of the covariance among all of our different measures using Harman's single factor test (see Podsakoff et al., 2003). As a result, four factors emerged, and the first method factor explained 43.15% of the variance. As several factors emerged, and the first method factor did not explain more than 50% of variance, we concluded, based on conventional standards, that common method bias was not a significant problem with our data and results.

We conducted a confirmatory factor analysis (CFA) to test for the distinctiveness of our core variables, namely, leader self-awareness, self-leadership, and leader self-efficacy. The fit indices were acceptable, although the CFI was slightly below the threshold: χ^2^ = 522.69 (*p* < 0.001), *df* = 167, CFI = 0.89, RMSEA = 0.07, and SRMR = 0.05. Overall, and as both SRMR and RMSEA were close to the suggested cut-off criteria (Hu and Bentler, 1998), we considered the fit results to be satisfactory to continue our analyses to test our hypotheses.

Results from Model 1 largely showed acceptable model fit with χ^2^ = 556.98 (*p* < 0.001), *df* = 185, CFI = 0.89, RMSEA = 0.07, and SRMR = 0.05. Moreover, all paths were related to each other as we had predicted. First, confirming Hypothesis 1, leader self-awareness was positively and significantly related to self-leadership with β = 0.40 (*SE* = 0.05), 95% CI (0.31; 0.49). Second, confirming Hypothesis 2, self-leadership positively related to leader self-efficacy of followers [β = 0.47 (*SE* = 0.05), 95% CI (0.37; 0.56)]. Concerning our control variable, gender did not relate to self-leadership significantly but did with leader self-efficacy [β = 0.09 (*SE* = 0.04), 95% CI (0.02; 0.17)], indicating that male participants felt higher levels of leader self-efficacy.

## Method Study 2

### Sample

We recruited the second sample via Amazon Mechanical Turk. We contacted participants about 2 weeks after they had finished the first survey to invite them to complete the second part. A total of *n* = 600 participants participated in the first survey, and a total of *n* = 411 completed both of our surveys. Another 56 individuals did not pass our attention checks, resulting in our final sample of *n* = 355 participants.

Participants in the second study had an age range between 22 and 75 years (*M* = 39.33, *SD* = 11.21). Most of our participants were male (57.7%). Only one person did not graduate from High School (0.3%), while another 4.8% stated that they did graduate from High School. Among those who went to College, 28 people (7.9%) stated that they had “some college” experience, while 31 (8.7%) had a 2-year degree, and as much as 193 people (54.4%) had a 4-year college degree. In addition to that, 21.1% had a professional degree and 2.5% a doctorate degree.

About a quarter of the participants worked up to 36 h (26.6%) a week, while another 50.5% worked between 36 and 40 h/week, and 12% worked between 40 and 45 h. The remaining 38 people worked up to 75 h/week. Participants worked in a broad range of industries, i.e., 56.3% worked in business or services, 11.8% in healthcare, 10.1% in education, and another 4.8% did labor work. Only 3.7% of the participants worked less than a year in their current organization, another 27.9% worked between 1 and 3 years, and 24.5% between 3 and 5 years. Another 40.6% had stayed with their company for more than 5 years. In terms of the followers' tenure with their current leader, 9.3% worked with their leader less than a year, while 67.8% had worked between 1 and 5 years, and another 22.9% worked with their current leader for more than 5 years.

### Measures and Analysis

Like in Study 1, we measured leader self-awareness, self-leadership, and leader self-efficacy. Additionally, we measured leadership emergence and nomination for promotion. Leader self-awareness and self-leadership were measured at Time 1, and leader self-efficacy, leadership emergence, and nomination for promotion were measured at Time 2. Again, we included gender as control variable. Additionally, we added a coronavirus disease (COVID)-related control variable, which will be described in more detail below.

We used the same measures for leader self-awareness (Avolio et al., 2018; α = 0.87), self-leadership (Houghton et al., 2012; α = 0.86), and leader self-efficacy (Hannah et al., 2012; α = 0.90) as in Study 1. Moreover, we measured leadership emergence with 4 items that were used by Lanaj and Hollenbeck (2015). We changed the wording from other ratings to self-ratings. A sample item is: “*I exhibit leadership.”* The scale had a Cronbach alpha of α = 0.93. Participants rated on a scale from 1 = *almost never* to 5 = *almost always*. Nomination for promotion was measured with 3 items. We used 2 items from Hoobler et al. (2009), adding the word “current:” “*My current manager would encourage me to apply for a promotion*” and “*My current manager has encouraged me to apply for a promotion*.” We added a third item, “*My previous manager encouraged me to apply for a promotion to a leadership position*,” to ensure we had a wider perspective on nomination for promotion, rather than just referring to the current manager. The scale was reliable with α = 0.86. Participants rated the items on a scale from 1 = *strongly disagree* to 7 = *strongly agree*.

In this study, we included a COVID-19-related control variable in addition to gender. We did so because we collected the data during the ongoing pandemic in summer 2020, while Study 1 was collected ~1 year earlier. The pandemic pushed many organizations and individuals in the US into a crisis situation (cf., Rotblut and Hageman, 2020), which we were concerned could bias data related to ratings of leadership and efficacy. Prior research has shown that an organizational performance crisis can impact the selection of leaders (Rink et al., 2013), in which women are more likely to be selected for leadership positions than men. In order to account for the pandemic and its associated disruptions impact on our study, we controlled for the COVID-related event disruption. We measured event disruption based on items developed by Morgeson (2005), which we adapted to fit the specific COVID context. A sample item is: “*To what extent has the coronavirus disrupted your ability to get your work done?*” The scale was reliable (α = 0.84). Answers were given on a 5-point Likert scale: 1 = *not at all*, 2 = *to a limited extent*, 3 = *to a moderate extent*, 4 = *to a large extent*, and 5 = *to a very large extent*. Event disruption was measured at Time 1.

Like in Study 1, we tested our hypotheses using structural equation modeling techniques with Mplus version 8 (Muthén and Muthén, 1998–2017). In this study, we tested all proposed hypotheses in one model simultaneously, including the two control variables. More precisely, we regressed both mediators and outcome variables on gender as well as on the latent COVID-disruption factor. We refer to the same fit indicators as described for Study 1 above.

## Results Study 2

Calculating means, standard deviations, and correlations for each scale construct, we found that all correlations between core variables were significant at *p* < 0.01, showing that leader self-awareness and self-leadership correlated with *r* = 0.26, self-leadership and leader self-efficacy with *r* = 0.53, leader self-efficacy and leadership emergence with *r* = 0.71, and finally, leader self-efficacy and nomination for promotion with *r* = 0.57. Exploring correlations with control variables, we found that gender correlated with leader self-efficacy (*r* = −0.11, *p* < 0.05), and leader emergence (*r* = −0.16, *p* < 0.01), such that male participants indicated higher levels of both leader self-efficacy and leadership emergence. COVID disruption positively related to all core variables at *p* < 0.01, varying between *r* = 0.15 and *r* = 0.30. All results can be found in Table 2.

**Table 2:** Means, standard deviations, and bivariate correlations for Study 2.

	**Mean (SD)**	**1**	**2**	**3**	**4**	**5**	**6**
Leader self-awareness (l)	3.51 (0.96)						
Self-leadership (f)	3.97 (0.66)	0.26^**^					
Leader self-efficacy (f)	5.35 (0.97)	0.34^**^	0.53^**^				
Leadership emergence (f)	3.52 (0.97)	0.33^**^	0.36^**^	0.71^**^			
Nomination for promotion (f)	4.85 (1.53)	0.53^**^	0.35^**^	0.57^**^	0.53^**^		
COVID-disruption (f)	3.05 (0.98)	0.17^**^	0.15^**^	0.30^**^	0.26^**^	0.20^**^	
Gender^a^ (f)	-	−0.10	0.05	−0.11^*^	−0.16^**^	−0.07	−0.06

** *p < 0.01,*

* *p < 0.05*.

*(l), leader-related variable; (f), follower-related variable*.

a *Male participants were coded as 1, female participants as 2*.

Results for discriminant validity testing can be found in Table 3. Our findings largely confirm that the constructs within our model were sufficiently different from each other. Yet, the squared correlation between leader emergence and leader self-efficacy is 0.01 higher than the AVE of leader self-efficacy. This might indicate concerns with discriminant validity.

**Table 3:** Discriminant validity in Study 2.

		**Self-leadership**	**Leader self-efficacy**	**Leader emergence**	**Nomination for promotion**
	**AVE**	***r*** **^2^**	***r*** **^2^**	***r*** **^2^**	***r*** **^2^**
Leader self-awareness	0.62	0.07	0.15	0.13	0.34
Self-leadership	0.43	-	0.33	0.15	0.12
Leader self-efficacy	0.58	0.33	-	0.59	0.38
Leader emergence	0.76	0.15	0.59	-	0.33
Nomination for promotion	0.70	0.12	0.38	0.33	-

*AVE, average variance extracted*.

Since we used a conservative measure to detect discriminant validity, we add another more recently introduced method to detect issues with discriminant validity, known as the CICFA method (Rönkkö and Cho, 2022). Results of the CICFA method can be interpreted as follows. If the upper 95% CI limit of the correlation between two measures is above 0.90, this indicates a problem with discriminant validity. Our results confirm that there was no significant problem with discriminant validity in Study 2, as the upper CI limit was below 0.90 in all cases (Rönkkö and Cho, 2022). For more detailed findings see Table 4.

**Table 4:** Upper confidence intervals of correlations between factors to test for discriminant validity.

	**Leader self-awareness**	**Self-leadership**	**Leader self-efficacy**	**Leader emergence**
Self-leadership	0.38			
Leader self-efficacy	0.48	0.66		
Leader emergence	0.47	0.49	0.82	
Nomination for promotion	0.67	0.45	0.69	0.65

*Values above 0.90 indicate a problem with discriminant validity*.

Like in Study 1, we tested for common method bias, using Harman's single factor test (see Podsakoff et al., 2003). In this study, six factors emerged, and the first method factor explained 37.24% of the variance. As several factors emerged, and the first method factor did not explain more than 50% of variance, common method bias may not have had a significant effect on our data and results. However, as noted by Podsakoff et al. (2003), this test does not necessarily rule out common method bias. Next, we conducted a CFA to test for the distinctiveness of our core variables, namely, leader self-awareness, self-leadership, leader self-efficacy, leadership emergence, and nomination for promotion. Results provided an acceptable fit with χ^2^ = 488.83 (*p* < 0.001), *df* = 314, CFI = 0.94, RMSEA = 0.04, and SRMR = 0.05, so we continued with hypotheses tests.

**Figure 2:**
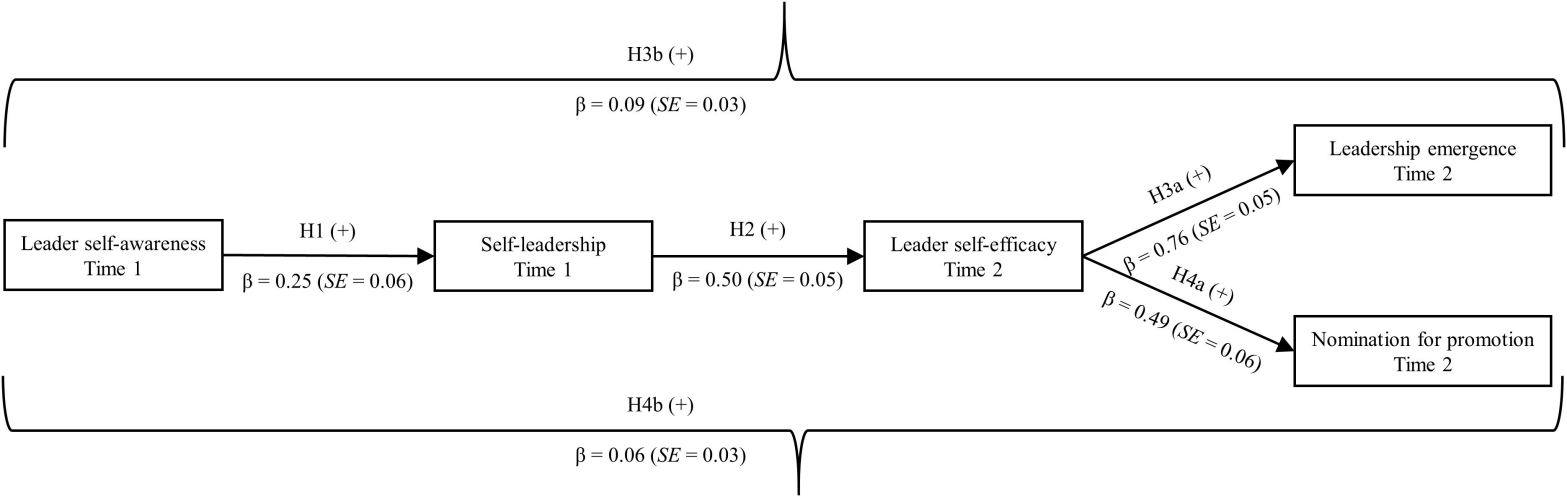
Model results of Study 2. All displayed relationships were significant at *p* ≤ 0.001.

The fit for the model testing the hypotheses was acceptable: χ^2^ = 667.08 (*p* < 0.001), *df* = 446, CFI = 0.92, RMSEA = 0.04, SRMR = 0.05. All direct paths were significant at the *p* ≤ 0.001 level. More precisely, leader self-awareness positively related to self-leadership with β = 0.25 (*SE* = 0.06), 95% CI (0.12; 0.37), and self-leadership positively related to leader self-efficacy with β = 0.50 (*SE* = 0.05), 95% CI (0.39; 0.60). Furthermore, leader self-efficacy was related to leadership emergence [β = 0.76 (*SE* = 0.05), 95% CI (0.66; 0.85] and nomination for promotion [β = 0.49 (*SE* = 0.06), 95% CI (0.37; 0.60)]. Lastly, leader self-awareness was related to leadership emergence through self-leadership and leader self-efficacy with β = 0.09 (*SE* = 0.03), 95% CI (0.04; 0.15), and nomination for promotion through the same mediators with β = 0.06 (*SE* = 0.03), 95% CI (0.02; 0.10). As the direct relationship between leader self-awareness and leadership emergence [β = 0.08 (*SE* = 0.04), 95% CI (−0.001; 0.17)] was insignificant, but the direct relationship between leader self-awareness and nomination for promotion [β = 0.42 (*SE* = 0.06), 95% CI (0.31; 0.54)] remained significant, when all mediators and control variables were in the model, we confirmed Hypothesis 3b but only partially confirmed Hypothesis 4b. Furthermore, we reconfirmed Hypotheses 1 and 2 and additionally accepted Hypotheses 3a and 4a. An overview of all results can be found in Figure 2.

Considering control variables, we first found that gender was related to leader self-efficacy [β = −0.11, *SE* = 0.04, 95% CI (−0.19; −0.02)], such that male participants experienced higher levels of leader self-efficacy, while gender did not relate to self-leadership, leadership emergence, or nomination for promotion. Second, event disruption was significantly related to leader self-efficacy [with β = 0.23 (*SE* = 0.05), 95% CI (0.13; 0.32)] but not to any of the other variables in the model. Interestingly, the more disruption participants experienced, the higher they rated their leader self-efficacy. This may be due in part to the timing of our study, in that for most participants, they were 3–4 months into the pandemic, where they may have developed specific strategies to mitigate the risks of the pandemic, for instance by working from home, reducing interactions outside one's so-called “bubble,” more available testing, and having a clearer sense of how the virus is transmitted. All of these factors could contribute to greater agency (Bandura, 2006). We address this finding in our future research and limitations section below.

## Discussion

The aim of the present study was to explore the role of a leader's level of leader self-awareness in triggering the follower's internal processes preceding the emergence of their leadership. Our findings confirmed a positive relationship between a leader's leader self-awareness and a follower's (a) leadership emergence and (b) nomination for promotion into a leadership position. Both relationships were shown to be serially mediated by the follower's self-leadership and the follower's leader self-efficacy, even after including controls for gender and COVID-disruption.

### Contribution to Research

We contribute to the leader(ship) emergence literature by providing evidence of how leaders can foster leadership emergence in their followers by developing their own level of leader self-awareness. Unlike previous work on antecedents of leadership emergence that primarily focused on follower attributes (Walter et al., 2012; Charlier et al., 2016; Kwok et al., 2018; Wyatt and Silvester, 2018), here, we include a mixture of internal and external variables to explain how leaders can emerge in response to their social context, namely, their leader's behavior. Thereby, we aimed to describe how leadership emergence may unfold over time, starting with leader self-awareness that triggers the follower's internal development processes toward self-leadership. This then guides the way toward the follower's own leadership emergence.

Doing so, we make an important contribution to the leader(ship) emergence literature, since to our best knowledge, most research in this area holds a rather static perspective on factors that determine leader and leadership emergence, and only a few studies (e.g., Reichard et al., 2011; Liu et al., 2019) have explored the pathways toward leader emergence. Yet, such process-oriented research is needed in order to gain a better understanding of how organizations and leaders can support the emergence of capable leaders. Therefore, a strength of our study is that by including leader self-awareness as a context variable, while combining it with self-leadership and leader self-efficacy as individual difference variables, we provided a more comprehensive model that encompasses the relationship between external and internal variables preceding leadership emergence. We consider this a strength of our research because it reflects that leadership emergence, just like most other phenomena in leadership, is an interactive process between individuals and context (e.g., Porter and McLaughlin, 2006; Avolio, 2007; Jepson, 2009; Oc, 2018), rather than solely determined by an individual's personal characteristics.

Furthermore, we contribute to the leadership literature by underscoring the importance leaders have for follower leadership development and emergence. Work on leadership has pointed out that the main task for leaders is to develop their followers into leaders themselves (Burns, 2012). Yet, despite the literature showing positive relations between positive forms of leadership and follower leadership (e.g., Schaubroeck et al., 2012), the process of how this unfolds largely remains untested (see also Siangchokyoo et al., 2020). While we did not intend to uncover the full transformation process from being a follower to becoming and emerging as a leader, we do contribute a small piece to understanding which dynamics may lie between a leader's behavior and a follower's internal processes toward becoming a leader.

Prior leader development research has also primarily examined the development of individual knowledge, skills, and ability (e.g., Mumford et al., 2007). Although the leadership field does recognize that development of leadership includes deeper transformative change, as well as a collective or shared process that evolves over time (DeRue and Myers, 2014), there has been very little empirical evidence provided on this transformation process. We call for future leadership research to seek greater clarity in terms of what could be developed based on understanding the dynamics between the social context, including one's leader, and how followers learn in that context to emerge as leaders.

Third, we contribute to the self-leadership literature by showing that leader self-awareness is a relevant antecedent for self-leadership. This adds to previous work, as other studies connecting leadership and follower self-leadership were largely focused on empowering leadership (Amundsen and Martinsen, 2014, 2015). Empowering leadership is “the process of influencing subordinates through power sharing, motivation support, and development support with intent to promote their experience of self-reliance, motivation, and capability to work autonomously within the boundaries of overall organizational goals and strategies” (Amundsen and Martinsen, 2014, p. 489). From this definition, we can see that empowering leadership has the goal to foster follower's autonomy and, hence, does relate to self-leadership in a very direct and explicit way. Leader self-awareness, however, taps into more implicit elements of self-leadership, which are inherent in some of the self-leadership strategies (Houghton and Neck, 2002). Thus, our findings contribute to broadening the understanding of how leadership can encourage followers' self-leadership emergence and development.

Lastly, an empirical strength of our study is that we tested parts of the mediation chain twice. The replication of our results underscores the relevance of our findings in the midst of the replication crisis emerging in psychology research (e.g., Lilienfeld, 2017; Shrout and Rodgers, 2018). Specifically, oftentimes an original study shows certain significant effects or relationships, while a second replication study does not confirm the same significant effects (Maxwell et al., 2015). Hence, by showing the same effects in two independent studies, we at least provide first evidence that the effects were not specific to one selected sample, which increases the level of robustness of our findings.

### Practical Implications

Our findings provide organizational leaders and developers with a strategy to examine how leaders can help their followers develop as leaders, which is by showing their own level of leader self-awareness and supporting their follower's successful self-leadership enactment. This is an important implication for organizations because it provides a pathway for organizations and leaders to prepare those individuals for leadership, who are potentially highly effective but otherwise may not have had the opportunity to emerge as leaders because of their more dominant (Hegstrom and Griffith, 1992) and narcissistic (Nevicka et al., 2011) counterparts who might squeeze them out of leadership roles in their organizations.

Our findings also provide implications for individuals who strive toward becoming a leader. These individuals may benefit from working on their own self-leadership first and then building on this competency and confidence to then take the next step to not just lead themselves but also actively seek roles to lead others (cf., Drucker, 1999; Lovelace et al., 2007; Furtner et al., 2013). On this journey, they may see their leader as an inspiration for self-awareness and a relevant supporter of their own self-leadership development.

### Limitations and Future Research

Although the time-lagged design of our study can be considered a strength compared to a cross-sectional study, it is also a clear limitation. Specifically, we theoretically focused on examining a longitudinal developmental process but then tested that process within a time span of only a few weeks. Hence, using the present study designs, we did not actually focus on, nor capture, the actual developmental process that ensued between the leader and their follower. Yet, as we theoretically derived the mediation chain, and included participants who were already leaders, we can see our findings as being a preliminary confirmation that such a developmental process might have occurred, resulting in higher levels of a follower's self-leadership and a follower's nomination for leadership promotion. In addition, although the approach certainly has its flaws, it is not unusual to test leadership-related interactive developmental processes with a comparable design (Fischer et al., 2017). Hence, building on our initial findings, future work may explore the mediation chain, or parts of it, within a longitudinal data set, capturing the changes and focusing on development as it unfolds over time.

A second limitation of our present research concerns the leader self-awareness—follower's self-leadership link. As described in the present research, there is significant overlap between self-awareness and self-leadership. Yet, while leader self-awareness is certainly important for followers to develop self-leadership, we can assume that it is not necessarily sufficient. Rather, a number of factors may come into play that determine if self-leadership can actually be practiced, such as urgency, need for creativity and innovation, as well as the degrees of interdependence, and complexity (Pearce and Manz, 2005). As our findings generally supported the positive relationship between leader self-awareness and follower's self-leadership, we suggest that future research may dive deeper into exploring this relationship as it unfolds over time. Thereby, we encourage the use of contextual moderators, which may shed more light on understanding under which conditions leader self-awareness might be especially strong, or less strongly related to follower's self-leadership, such as in climates where there is a higher degree of psychological safety and voice.

While we did measure nomination for promotion and leadership emergence as outcome variables, we captured them as self-ratings and did not include other-rated measures of the emerging leader or in the form of leadership effectiveness (e.g., Wang et al., 2018). These might be important variables to finally determine whether self-leadership and leader self-efficacy actually lead to the emergence of better and more effective leaders. Therefore, while we can theoretically assume such a positive connection, future work should explore not just *if* leaders can emerge from the process described in this work but also *how* these leaders behave, for instance by measuring their leadership behavior and performance with a range of different leadership measures (Northouse, 2018).

Building on our finding that leaders can encourage their follower's leadership emergence, future work might focus on other forms and elements of leadership, as well as followership, and how they relate to leadership emergence. For example, empowering leadership (Amundsen and Martinsen, 2014), a form of leadership that actively encourages the follower to act autonomously and take on responsibility, as well as transformational leadership (Avolio and Bass, 2004), which aims to develop followers into leaders (Burns, 2012), may serve as interesting starting point for such future research.

In terms of the potential impact of the pandemic on our participants in Study 2, Bandura (1971, 1986) suggests that when individuals take on more challenging tasks early in the learning process, in our case, the early stages of the pandemic unfolding, they are more likely to be build a greater sense of efficacy if successful than when they engage in less challenging tasks. This might partially explain why the impact of the COVID 19 pandemic related positively to a follower's level of leader self-efficacy.

Future research should also now consider exploring not only when individuals take on these types of consequential challenges but also the type and/or characteristics of the consequential event/crisis. For example, some consequential events/crises may have more of an impact on financial or reputational losses, as opposed to the pandemic impact on mortality rates. Thus, the nature of the consequential event/crisis may matter to how the leader's effectiveness is viewed. Moreover, other research on the COVID 19 pandemic has found that there may also be gender differences in terms of how leaders interact with their key stakeholders/constituents when responding to this crisis. For example, Sergent and Stajkovic (2020) reported in their investigation of governors in the US responding to the COVID 19 pandemic that women vs. male governors had fewer COVID 19 deaths in their states. Moreover, based on a qualitative analysis of the governor's speeches during the pandemic, the authors concluded that women governors expressed more empathy and confidence to their constituents, as they navigated through this crisis. Examining gender differences in how leaders respond to different consequential events seems like a fruitful area for future leadership research.

### Conclusion

In the present work, we showed that leader self-awareness was positively associated with the follower's leadership emergence through the follower's self-leadership and leader self-efficacy. These findings are encouraging in that they underline the idea that there are positive pathways to leadership emergence in the form of stepwise development toward leadership emergence. This gives us hope that when leaders and organizations recognize such alternative pathways to leadership emergence and actively work on supporting them, they can create more opportunities for individuals with diverse educational, gender, race, and ethnicity backgrounds to emerge as leaders based on their capability to lead well and do good for their respective organizations.

## Data Availability Statement

The raw data supporting the conclusions of this article will be made available by the authors, without undue reservation.

## Ethics Statement

For Study 1 ethical review and approval was not required for the study on human participants in accordance with the local legislation and institutional requirements. The participants provided their written informed consent to participate in this study. Study 2 involving human participants was reviewed and approved by Nanyang Technological University (NTU) Institutional Review Board (IRB-2020-04-004). The participants provided their written informed consent to participate in this study.

## Author Contributions

All authors contributed to the article and approved the submitted version.

## Funding

The data collection in Study 1 was funded by the Willkomm Stiftung and the Department of Social Psychology at the Goethe University in Frankfurt, Germany together with the Center for Leadership and Strategic Thinking at the University of Washington in Seattle, Washington, USA. The data collection Study 2 was funded by the Center for Leadership and Strategic Thinking at the University of Washington in Seattle, Washington, USA, together with Nanyang Technological University in Nanyang, Singapore. Publishing fees are funded by the Goethe University, Frankfurt, Germany.

## Conflict of Interest

The authors declare that the research was conducted in the absence of any commercial or financial relationships that could be construed as a potential conflict of interest.

## Footnotes

^1^To increase readability, we will henceforth refer to a leader's leader self-awareness as leader self-awareness when describing parts of our model. All other variables of the model are follower related and will also be referred to as self-leadership, leader self-efficacy, leadership emergence, and nomination for promotion, without necessarily always including “follower's”.
