# Corrigendum: Take a “Selfie”: Examining How Leaders Emerge From Leader Self-Awareness, Self-Leadership, and Self-Efficacy

**DOI:** 10.3389/fpsyg.2021.745910

**Published:** 2021-08-23

**Authors:** Eva M. Bracht, Fong T. Keng-Highberger, Bruce J. Avolio, Yiming Huang

**Affiliations:** ^1^Department of Social Psychology, Institute of Psychology, Goethe University, Frankfurt, Germany; ^2^Nanyang Business School, Nanyang Technological University, Singapore, Singapore; ^3^Foster School of Business, University of Washington, Seattle, WA, United States; ^4^School of Business, Nanjing University, Nanjing, China

**Keywords:** information processing theory, leadership emergence, leader self-awareness, leader self-efficacy, self-leadership, social cognitive theory

In the original article, there was an error. The Ethics Statement incorrectly stated that “Ethical review and approval was not required for the study on human participants in accordance with the local legislation and institutional requirements. The patients/participants provided their written informed consent to participate in this study.”

A correction has been made to the Ethics Statement. The corrected statement is shown below.

The studies involving human participants were reviewed and approved by Nanyang Technological University (NTU) Institutional Review Board (IRB-2020-04-004). The participants provided their written informed consent to participate in this study.

The authors apologize for this error and state that this does not change the scientific conclusions of the article in any way. The original article has been updated.

## Publisher's Note

All claims expressed in this article are solely those of the authors and do not necessarily represent those of their affiliated organizations, or those of the publisher, the editors and the reviewers. Any product that may be evaluated in this article, or claim that may be made by its manufacturer, is not guaranteed or endorsed by the publisher.

