# Corrigendum: Take a “selfie”: Examining how leaders emerge from leader self-awareness, self-leadership, and self-efficacy

**DOI:** 10.3389/fpsyg.2022.913892

**Published:** 2022-10-06

**Authors:** Eva M. Bracht, Fong T. Keng-Highberger, Bruce J. Avolio, Yiming Huang

**Affiliations:** ^1^Department of Social Psychology, Institute of Psychology, Goethe University, Frankfurt, Germany; ^2^Nanyang Business School, Nanyang Technological University, Singapore, Singapore; ^3^Foster School of Business, University of Washington, Seattle, WA, United States; ^4^School of Business, Nanjing University, Nanjing, China

**Keywords:** information processing theory, leadership emergence, leader self-awareness, leader self-efficacy, self-leadership, social cognitive theory

In the original article, there was a mistake in Table 2 as published. The mean and standard deviation of leadership emergence needed to be changed from “4.85 (1.53)” to “3.52 (0.97)” and nomination for promotion had to be changed from “3.52 (0.97)” to “4.85 (1.53).” These were reversed. The correlation of gender and leadership emergence (column 4) was reported as “−16” but should have been “−0.16.” The updated Table 2 appears below.

**Table 2:** Means, standard deviations, and bivariate correlations for Study 2.

	**Mean (SD)**	**1**	**2**	**3**	**4**	**5**	**6**
Leader self-awareness (l)	3.51 (0.96)						
Self-leadership (f)	3.97 (0.66)	0.26^**^					
Leader self-efficacy (f)	5.35 (0.97)	0.34^**^	0.53^**^				
Leadership emergence (f)	3.52 (0.97)	0.33^**^	0.36^**^	0.71^**^			
Nomination for promotion (f)	4.85 (1.53)	0.53^**^	0.35^**^	0.57^**^	0.53^**^		
COVID-disruption (f)	3.05 (0.98)	0.17^**^	0.15^**^	0.30^**^	0.26^**^	0.20^**^	
Gender^a^ (f)	-	−0.10	0.05	−0.11^*^	−0.16^**^	−0.07	−0.06

** p < 0.01,

* p < 0.05.

(l), leader-related variable; (f), follower-related variable.

a Male participants were coded as 1, female participants as 2.

In **Method Study 1**, *Measures*, Paragraph 3, the word “one-dimensional” in “a nine-item one-dimensional measure of self-leadership” was incorrectly included. The paragraph has now been amended as follows:

“Self-leadership was captured using the *Abbreviated Self-Leadership Questionnaire* (Houghton et al., 2012), which is a nine-item measure of self-leadership. A sample item was: “*I try to mentally evaluate the accuracy of my own beliefs about situations I am having problems with.”* Cronbach alpha was α = 0.90. Participants rated the items on a scale from 1 = *strongly disagree* to 5 = *strongly agree*.”

In **Results Study 1**, Paragraph 2, *r*^2^ was incorrectly written as *R*^2^. Also, for the original discriminant analyses in this paragraph, we used factor loadings from exploratory factor analysis (EFA) results. We now updated this to using confirmatory factor analysis (CFA) for calculating factor loadings for the average variance extracted (AVE). We did so as in recent reviews examining the AVE method to test for discriminant validity, it is noted that the CFA is the most common technique used (Rönkkö and Cho, 2022). We updated the AVE and *r*^2^ values for Study 1 with minor changes. The paragraph has now been amended as follows:

“To determine whether our measures were sufficiently different from each other, we tested them for their discriminant validity. Discriminant validity can be confirmed to the degree that a latent variable explains a higher amount of variance in its indicator variables than it shares variance with other constructs (Fornell and Larcker, 1981). This criterion is met if the average variance extracted (AVE) regarding the focal factor is higher than its *r*^2^ with other factors (Henseler et al., 2015). Based on this criterion, we compared the AVE values of each construct in the model with its squared correlations with the remaining constructs. Results show that the AVE value for leader self-awareness was 0.72, which was higher than its squared correlations with self-leadership (*r*^2^ = 0.16) and leader self-efficacy (*r*^2^ = 0.08). Moreover, the AVE for self-leadership was 0.54 and was higher than its squared correlations with leader self-awareness and leader self-efficacy (*r*^2^ = 0.26). Finally, the AVE for leader self-efficacy was 0.73 and was higher than the squared correlations with leader self-awareness and self-leadership. Hence, we could confirm discriminant validity for all constructs in this study.”

In **Results Study 1**, Paragraph 4, the calculation of the value of χ^2^ and *df* were incorrectly reported. They were given as “χ^2^ = 3,518.27 (*p* < 0.001), *df* = 190” but should be “χ^2^ = 522.69 (*p* < 0.001), *df* = 167.” The paragraph has now been amended as follows:

“We conducted a confirmatory factor analysis (CFA) to test for the distinctiveness of our core variables, namely, leader self-awareness, self-leadership, and leader self-efficacy. The fit indices were acceptable, although the CFI was slightly below the threshold: χ^2^ = 522.69 (*p* < 0.001), *df* = 167, CFI = 0.89, RMSEA = 0.07, and SRMR = 0.05. Overall, and as both SRMR and RMSEA were close to the suggested cut-off criteria (Hu and Bentler, 1998), we considered the fit results to be satisfactory to continue our analyses to test our hypotheses.”

In **Method Study 2**, *Sample*, Paragraph 3, the means and standard deviations for the variable concerning the length participants had stayed with their current organization and worked with their current leader were incorrectly included because both variables are categorical variables. Both have been removed. In the same paragraph we originally reported that the % who had stayed with their company for more than 5 years was 22.8% and this should have been reported as 40.6%. The paragraph now reads as:

“About a quarter of the participants worked up to 36 h (26.6%) a week, while another 50.5% worked between 36 and 40 h/week, and 12% worked between 40 and 45 h. The remaining 38 people worked up to 75 h/week. Participants worked in a broad range of industries, i.e., 56.3% worked in business or services, 11.8% in healthcare, 10.1% in education, and another 4.8% did labor work. Only 3.7% of the participants worked less than a year in their current organization, another 27.9% worked between 1 and 3 years, and 24.5% between 3 and 5 years. Another 40.6% had stayed with their company for more than 5 years. In terms of the followers' tenure with their current leader, 9.3% worked with their leader less than a year, while 67.8% had worked between 1 and 5 years, and another 22.9% worked with their current leader for more than 5 years.”

In **Method Study 2**, *Measures and Analysis*, Paragraph 3, the value of α was incorrect. It was stated as “α = 0.86” but should be “α = 0.84.” The paragraph has now been amended as follows:

“In this study, we included a COVID-19-related control variable in addition to gender. We did so because we collected the data during the ongoing pandemic in summer 2020, while Study 1 was collected ~1 year earlier. The pandemic pushed many organizations and individuals in the US into a crisis situation (cf., Rotblut and Hageman, 2020), which we were concerned could bias data related to ratings of leadership and efficacy. Prior research has shown that an organizational performance crisis can impact the selection of leaders (Rink et al., 2013), in which women are more likely to be selected for leadership positions than men. In order to account for the pandemic and its associated disruptions impact on our study, we controlled for the COVID-related event disruption. We measured event disruption based on items developed by Morgeson (2005), which we adapted to fit the specific COVID context. A sample item is: “*To what extent has the coronavirus disrupted your ability to get your work done?*” The scale was reliable (α = 0.84). Answers were given on a 5-point Likert scale: 1 = *not at all*, 2 = *to a limited extent*, 3 = *to a moderate extent*, 4 = *to a large extent*, and 5 = *to a very large extent*. Event disruption was measured at Time 1.”

In **Results Study 2**, Paragraph 2, we updated the discriminant validity analyses like we did for Study 1 and adjusted Table 3. As the updated results might indicate concerns with discriminant validity for leader emergence and leader self-efficacy, we replaced the paragraph with the following paragraphs and Table 4, adding an additional analysis for discriminant validity.

**Table 3:** Discriminant validity in Study 2.

		**Self-leadership**	**Leader self-efficacy**	**Leader emergence**	**Nomination for promotion**
	**AVE**	***r*** **^2^**	***r*** **^2^**	***r*** **^2^**	***r*** **^2^**
Leader self-awareness	0.62	0.07	0.15	0.13	0.34
Self-leadership	0.43	-	0.33	0.15	0.12
Leader self-efficacy	0.58	0.33	-	0.59	0.38
Leader emergence	0.76	0.15	0.59	-	0.33
Nomination for promotion	0.70	0.12	0.38	0.33	-

AVE, average variance extracted.

**Table 4:** Upper confidence intervals of correlations between factors to test for discriminant validity.

	**Leader self-awareness**	**Self-leadership**	**Leader self-efficacy**	**Leader emergence**
Self-leadership	0.38			
Leader self-efficacy	0.48	0.66		
Leader emergence	0.47	0.49	0.82	
Nomination for promotion	0.67	0.45	0.69	0.65

Values above 0.90 indicate a problem with discriminant validity.

“Results for discriminant validity testing can be found in Table 3. Our findings largely confirm that the constructs within our model were sufficiently different from each other. Yet, the squared correlation between leader emergence and leader self-efficacy is 0.01 higher than the AVE of leader self-efficacy. This might indicate concerns with discriminant validity.”

“Since we used a conservative measure to detect discriminant validity, we add another more recently introduced method to detect issues with discriminant validity, known as the CICFA method (Rönkkö and Cho, 2022). Results of the CICFA method can be interpreted as follows. If the upper 95% CI limit of the correlation between two measures is above 0.90, this indicates a problem with discriminant validity. Our results confirm that there was no significant problem with discriminant validity in Study 2, as the upper CI limit was below 0.90 in all cases (Rönkkö and Cho, 2022). For more detailed findings see Table 4.”

In **Results Study 2**, Paragraph 3, explanation of the potential for common method to exist as referenced by Podsakoff et al. (2003) was missing. In Sentence 2 we have amended “indicating that common method bias did not necessarily impact the interpretation of our results” to “As several factors emerged, and the first method factor did not explain more than 50% of variance, common method bias may not have had a significant effect on our data and results. However, as noted by Podsakoff et al. (2003), this test does not necessarily rule out common method bias.” We also corrected the values of χ^2^, df, CFI, RMSEA, and SRMR. They were given as χ^2^ = 2,848.08 (*p* < 0.001), df = 351, CFI = 0.90, RMSEA = 0.05, and SRMR = 0.08” but should be “χ^2^ = 488.83 (*p* < 0.001), df = 314, CFI = 0.94, RMSEA = 0.04, and SRMR = 0.05.” The paragraph has now been amended as follows:

“Like in Study 1, we tested for common method bias, using Harman's single factor test (see Podsakoff et al., 2003). In this study, six factors emerged, and the first method factor explained 37.24% of the variance. As several factors emerged, and the first method factor did not explain more than 50% of variance, common method bias may not have had a significant effect on our data and results. However, as noted by Podsakoff et al. (2003), this test does not necessarily rule out common method bias. Next, we conducted a CFA to test for the distinctiveness of our core variables, namely, leader self-awareness, self-leadership, leader self-efficacy, leadership emergence, and nomination for promotion. Results provided an acceptable fit with χ^2^ = 488.83 (*p* < 0.001), *df* = 314, CFI = 0.94, RMSEA = 0.04, and SRMR = 0.05, so we continued with hypotheses tests.”

In the article the **Ethics Statement** gave information for Study 2 but not for Study 1. The revised statement appears below.

The authors apologize for these errors and state that they do not change the scientific conclusions of the article in any way. The original article has been updated.

## Ethics statement

For Study 1 ethical review and approval was not required for the study on human participants in accordance with the local legislation and institutional requirements. The participants provided their written informed consent to participate in this study. Study 2 involving human participants was reviewed and approved by Nanyang Technological University (NTU) Institutional Review Board (IRB-2020-04-004). The participants provided their written informed consent to participate in this study.

## Publisher's note

All claims expressed in this article are solely those of the authors and do not necessarily represent those of their affiliated organizations, or those of the publisher, the editors and the reviewers. Any product that may be evaluated in this article, or claim that may be made by its manufacturer, is not guaranteed or endorsed by the publisher.
